# Flexible Bayesian longitudinal models for cost-effectiveness analyses with informative missing data

**DOI:** 10.1002/hec.4408

**Published:** 2021-09-25

**Authors:** Alexina J. Mason, Manuel Gomes, James Carpenter, Richard Grieve

**Affiliations:** 1 Department of Health Services Research and Policy, LSHTM, University of London, London, UK; 2 Department of Applied Health Research, University College London, London, UK; 3 Department of Medical Statistics, LSHTM, University of London, London, UK; 4 MRC Clinical Trials Unit at UCL, London, UK

**Keywords:** Bayesian analysis, cost-effectiveness analysis, missing not at random, selection model, sensitivity analysis

## Abstract

Cost-effectiveness analyses (CEA) are recommended to include sensitivity analyses which make a range of contextually plausible assumptions about missing data. However, with longitudinal data on, for example, patients’ health-related quality of life (HRQoL), the missingness patterns can be complicated because data are often missing both at specific timepoints (interim missingness) and following loss to follow-up. Methods to handle these complex missing data patterns have not been developed for CEA, and must recognize that data may be missing not at random, while accommodating both the correlation between costs and health outcomes and the non-normal distribution of these endpoints. We develop flexible Bayesian longitudinal models that allow the impact of interim missingness and loss to follow-up to be disentangled. This modeling framework enables studies to undertake sensitivity analyses according to various contextually plausible missing data mechanisms, jointly model costs and outcomes using appropriate distributions, and recognize the correlation among these endpoints over time. We exemplify these models in the REFLUX study in which 52% of participants had HRQoL data missing for at least one timepoint over the 5-year follow-up period. We provide guidance for sensitivity analyses and accompanying code to help future studies handle these complex forms of missing data.

## 1 Introduction

International methods guidance for cost-effectiveness analyses (CEA) requires evidence about treatment effectiveness from well-designed randomized controlled trials (RCTs) (Sanders et al., 2016). Many CEA use RCT evidence about patients’ health-related quality of life (HRQoL), measured at regular timepoints during the trial follow-up period. A common problem is that some of these longitudinal data are missing, as patients are lost to follow-up, or fail to complete the requisite questionnaires at each timepoint (Gabrio et al., 2017; Leurent et al., 2018a). Methods guidance requires the study to consider the uncertainty pertaining to the missing data mechanism (Faria et al., 2014; Leurent et al., 2018b). CEA often assume that the missingness only depends on the observed data, in that the data are “missing at random” (MAR) (Gabrio et al., 2017; Leurent et al., 2018a). However, in many settings, missing data may depend on outcomes that are unobserved, for example, the patients’ health status, and it is more reasonable to assume the data are “missing not at random” (MNAR) (Leurent et al., 2020; Mason et al., 2018). The “true” underlying missing data mechanism cannot be verified from the data at hand, and hence CEA are recommended to report sensitivity analyses according to alternative assumptions about missing data (Faria et al., 2014; Leurent et al., 2018b, 2020; Mason et al., 2018).

CEA with longitudinal data tend to have complex patterns of missing data which take several forms (Faria et al., 2014; Gabrio et al., 2020). For example, participants may be lost to follow-up, so that no further outcome data are available for that individual, or they may remain within the study, but fail to provide complete data at particular timepoints within the follow-up period (interim missingness). The approach to the missing data should then recognize that the reasons for loss to follow-up versus interim missingness may be different. Previous approaches to handling MNAR data in CEA have focused on pattern-mixture models and not considered these different forms of missing data within the longitudinal setting (Faria et al., 2014; Leurent et al., 2018b, 2020; Mason et al., 2018). Pattern-mixture models formulate the MNAR problem in terms of different distributions between missing and observed data. However, as these studies recognized, pattern-mixture models are less attractive to handle MNAR in longitudinal studies. For example, such forms of pattern-mixture model require strong assumptions about the differences between the observed and missing data distributions (sensitivity parameters) for each timepoint and do not readily allow the analyst to make plausible assumptions about the different forms of missing data.

Selection models offer an appealing approach to formulating the requisite sensitivity analyses in studies faced with different forms of missing data across multiple timepoints, and they have been applied within simple settings in comparative effectiveness research (Daniels & Hogan, 2008; Mason et al., 2012; Molenberghs et al., 2015). However, CEA raises additional challenges for the application of selection models for handling the missing data. First, costs and health outcomes tend to be correlated and need to be modeled jointly (Grieve et al., 2010; Nixon & Thompson, 2005; O’Hagan & Stevens, 2001). Second, CEA endpoints tend to have non-normal distributions, which complicates the MNAR modeling. In particular, considerable attention in the health econometrics literature has been given to developing models that recognize that HRQoL are left-skewed with spikes at 1, but these models have not been extended to common settings with missing data (Basu & Manca, 2012; Gomes et al., 2019; Hernandez-Alava et al., 2012). Modeling CEA endpoints according to plausible distributional assumptions is important in selection approaches because these directly identify the distribution of the unobserved values conditional on the observed data. Hence, currently available methods do not address fundamental concerns that arise when using longitudinal data in CEA.

Bayesian methods can help CEA provide evidence for directly informing decision-making, while allowing for complexities, such as the correlations between costs and health outcomes, the longitudinal structure in the data, and the need to make appropriate distributional assumptions (Baio, 2013; Lambert et al., 2008; Nixon & Thompson, 2005). Bayesian frameworks for addressing missing data in CEA have been proposed, but do not address the common challenges that arise with longitudinal data. The aim of this paper is to develop a Bayesian approach to CEA with longitudinal data, which uses selection models to make different, plausible assumptions about the missing data mechanism. This approach to sensitivity analyses is flexible, in that it can recognize different reasons for the missing outcome data at each timepoint, specify appropriate distributional assumptions for the costs and outcomes, and acknowledge the correlation between the endpoints. We exemplify our approach by re-analyzing the REFLUX study, which has a substantial proportion of patients (over 50%) with HRQoL data missing for at least one timepoint over the 5-year follow-up (Grant et al., 2013).

The remainder of this paper is structured as follows. Next we give more details on REFLUX, our motivating example that illustrates the challenges faced by CEA of longitudinal studies with MNAR data, and then we describe the proposed selection model framework. We then describe the development of the models through the re-analysis of the REFLUX case study, and present the results. The discussion highlights the main strengths and limitations of the proposed selection model approach and discusses some avenues for further research. Software code (using R and JAGS) is provided as supplementary material.

## 2 Methods

### 2.1 Motivating example: the REFLUX trial

This CEA used information from an RCT in which 357 patients with moderately severe gastroesophageal reflux disease recruited from 21 hospitals were randomly assigned to laparoscopic surgery (LS) (*n* = 178), or medical management (MM) (*n* = 179) (Grant et al., 2013). QALYs were calculated from responses to the EQ-5D-3L questionnaire administered at baseline, 3 months and annually for 5 years post-randomization. Annual costs were collected and combined with QALYs to report estimates of relative cost-effectiveness over 5 years. In the base case analyses presented in the primary study publications, missing data were addressed using multiple imputation assuming MAR (Grant et al., 2013). The authors also presented a complete case analysis (CCA), and an MNAR sensitivity analysis that assumed patients with missing data at a particular follow-up timepoint had relatively low HRQoL, or high costs. The base case analysis and CCA suggested that LS was cost-effective relative to MM. The findings of the MNAR sensitivity analyses were somewhat mixed; the results were insensitive to alternative assumptions about the missing costs, but appeared less robust to different assumptions about the missing HRQoL data. In particular, the authors state:

the cost-effectiveness of surgery is highly sensitive if it is assumed that surgery-allocated patients with missing data experience lower HRQoL than patients with complete data (Grant et al., 2013, p. 75).

**Figure 1:**
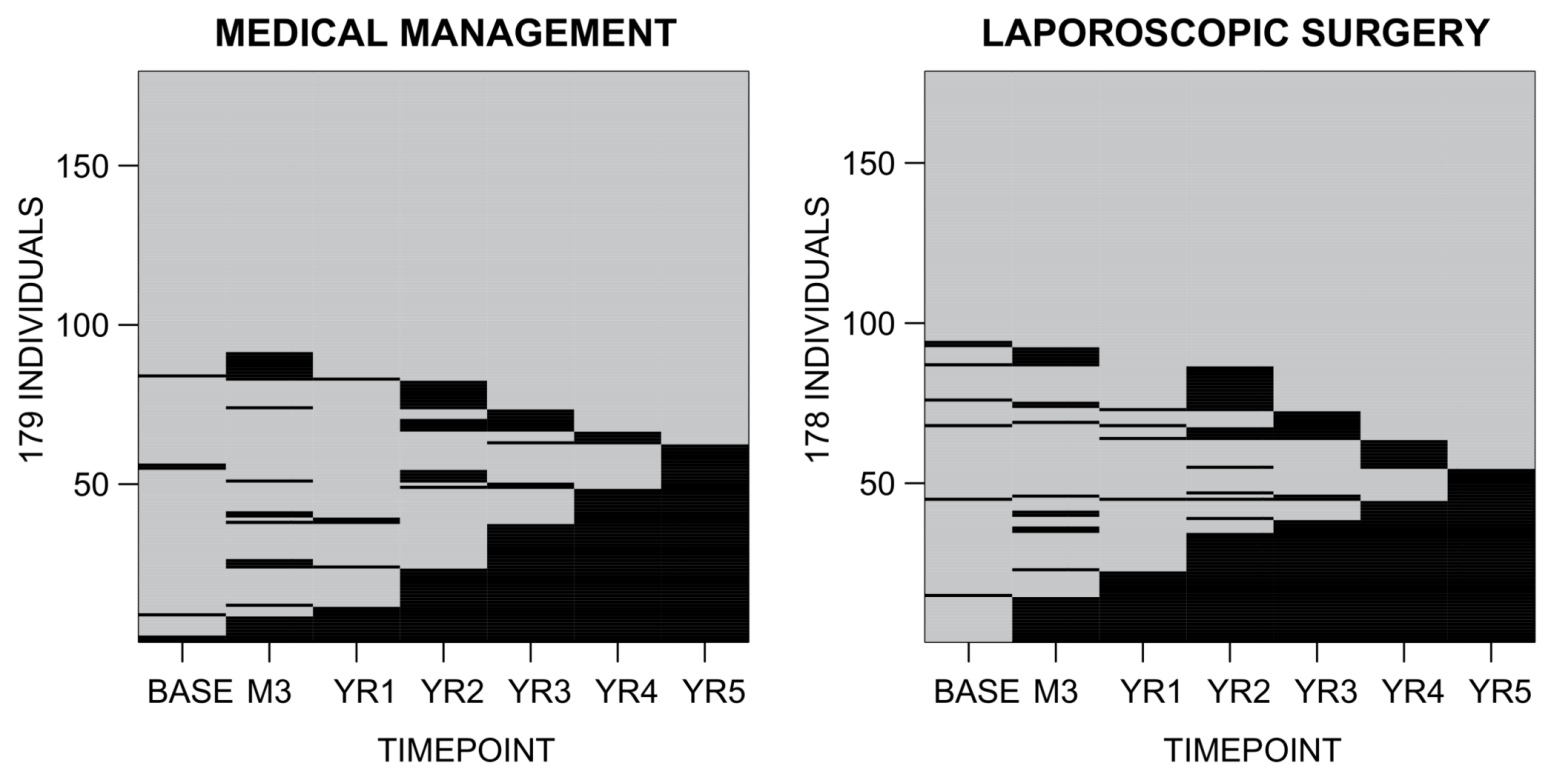
Pattern of missing health-related quality of life (HRQoL) by treatment arm. Black shading represents missing HRQoL for individuals (vertical axis) by timepoint (horizontal axis); gray shading represents observed HRQoL.

**Figure 2:**
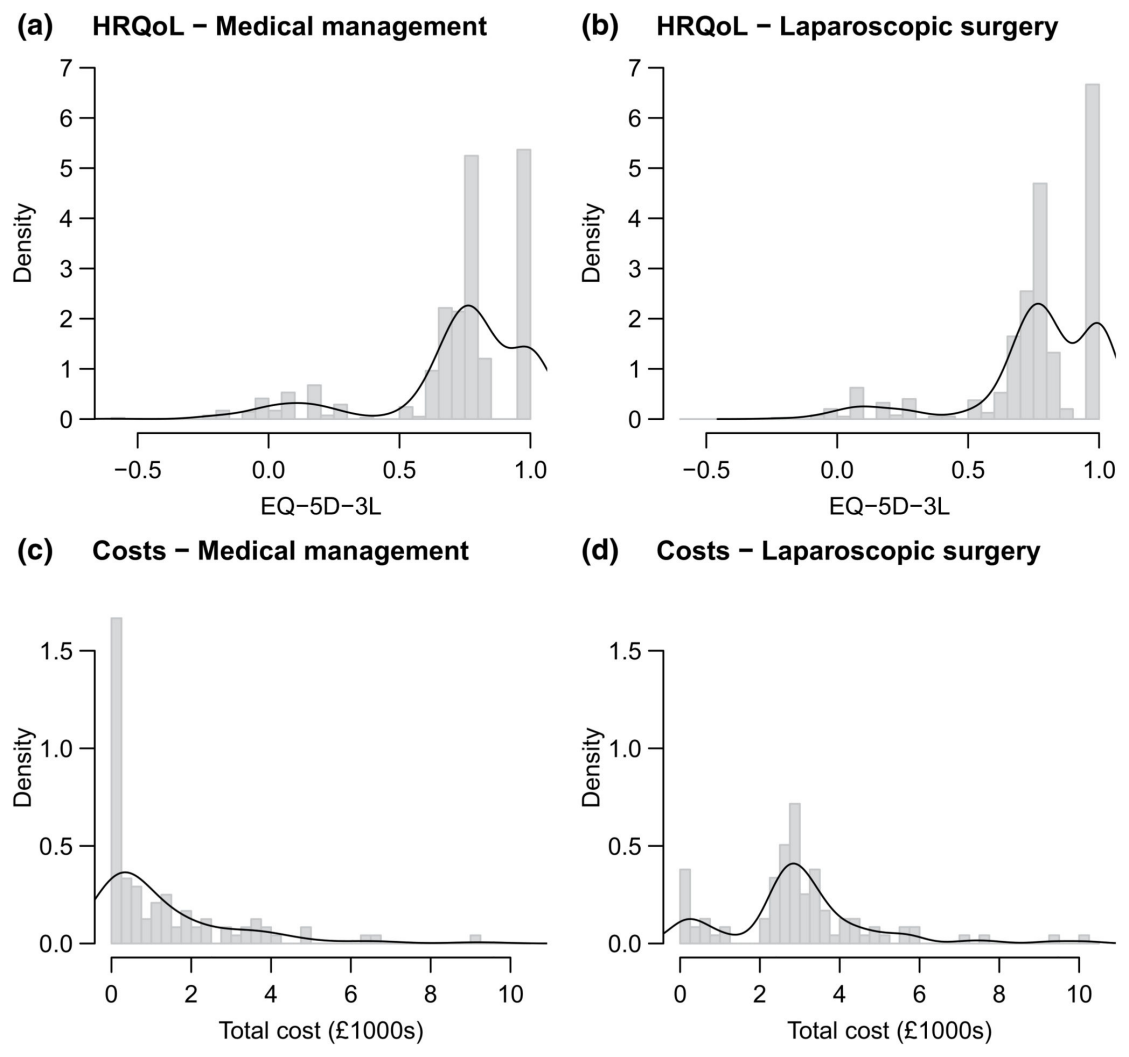
Distribution of health-related quality of life (HRQoL) (top) and costs (bottom) by treatment arm. An estimated kernal density has been superimposed on each histogram. HRQoL is across all timepoints (3 months and years 1–5)

While the REFLUX study did, therefore, consider the implications of missing data for the study conclusions, more flexible analytical approaches are required to address several related challenges that commonly occur with longitudinal HRQoL data. First, across the 5-year follow-up period, there are different forms of missing HRQoL data: 19.3% of patients have interim missing data, 24.9% loss to follow-up, and 7.6% have interim missingness, and then loss to follow-up. Figure 1 provides a more detailed representation of the missing HRQoL data patterns, and compares study arms. Second, LS is a “one-off” intervention, whereas MM could be provided throughout the follow-up period, and so the reasons for missing HRQoL data are likely to differ by treatment strategy. Third, the alternative forms of missing data may arise for different reasons; patients may be more inclined to “drop-out” following a large change in health status, whereas “interim” missingness could be “uninformative”, that is unrelated to health status, or to reflect a temporary change in health or circumstances. Fourth, neither HRQoL nor costs are normally distributed (Figure 2). Fifth, total QALYs and total costs are correlated (correlation coefficient of −0.42 for the group assigned to MM, −0.07 for LS). Sixth, the study faced the common challenge of crossover, in that 67 (37.6%) patients randomized to LS received MM, and 10 (5.6%) patients randomized to MM received LS.

Motivated by these common concerns for CEA that use longitudinal data, we now propose a flexible Bayesian approach to handling missing data within the longitudinal setting.

### 2.2 Proposed approach

#### 2.2.1 Bayesian selection model overview

**Figure 3:**
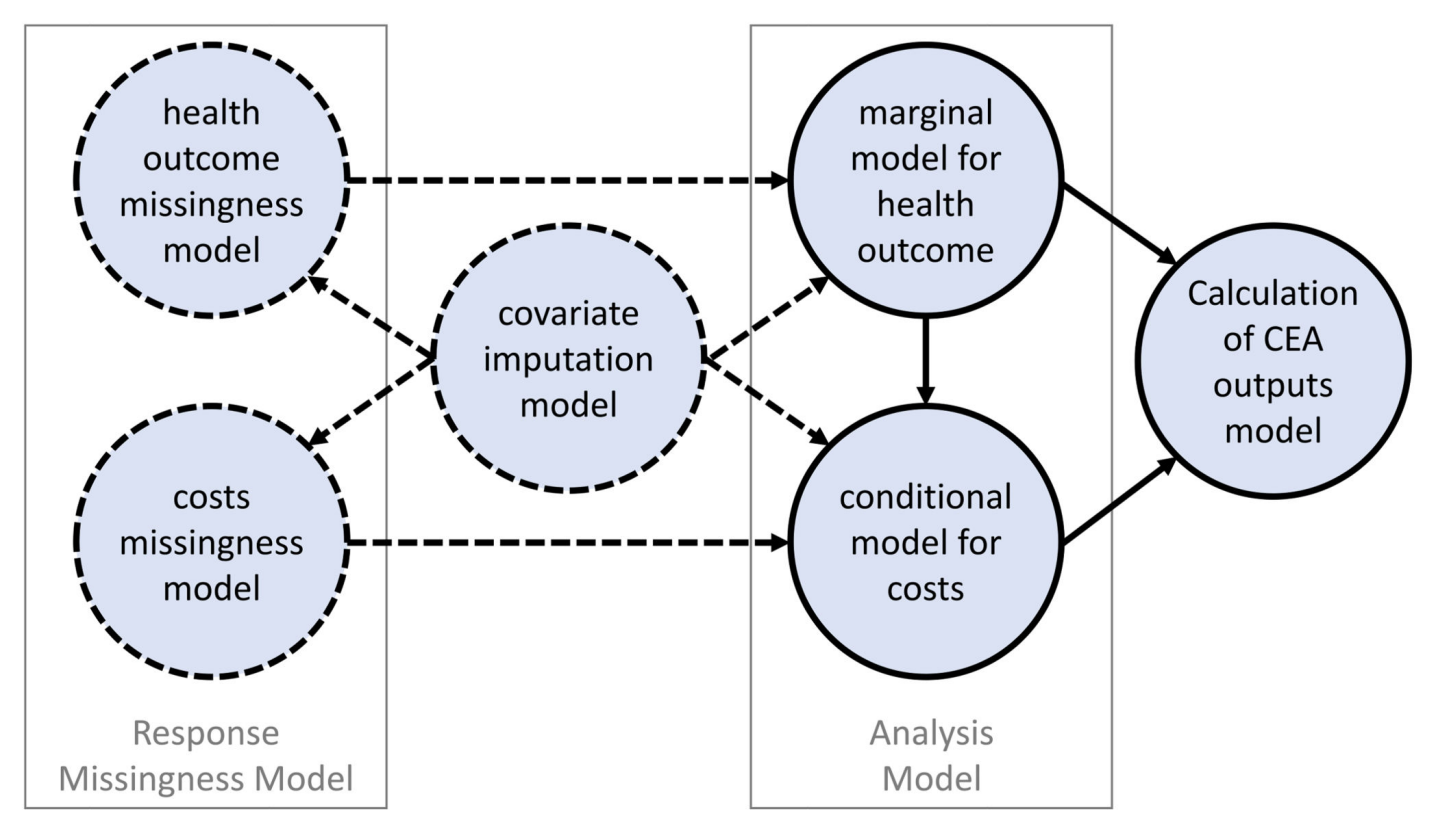
Schematic diagram of a typical Bayesian joint model for cost-effectiveness analysis. The sub-models shown with a solid outline will always be required, the requirement for those with a dashed outline depends on which variables have missing values and the assumptions about the missingness mechanism [Colour figure can be viewed at wileyonlinelibrary.com]

We build on previous Bayesian methods for CEA (Baio, 2013, 2014; Gabrio et al., 2019, 2020; Grieve et al., 2010; Nixon & Thompson, 2005; O’Hagan & Stevens, 2001; Thompson & Nixon, 2005). We propose a Bayesian longitudinal selection model, which contains sub-models to handle the important complexities raised by missing data within the longitudinal setting. This approach harnesses the computational power and flexibility of Markov Chain Monte Carlo methods to undertake analyses that make different assumptions in addressing these complexities, within a single modeling framework. This model estimates a substantive model for the CEA endpoints (analysis model) and a model for the missingness (missingness model). Figure 3 shows the links between the various sub-models.

For ease of implementation, the joint analysis model for the health outcome and costs is specified as a marginal model for the health outcome and a conditional model for the costs. The three sub-models shown with a solid outline are fundamental for the CEA, but not all those with a dashed outline are necessarily required. This model could be further extended to allow for MNAR covariate missingness by adding a covariate missingness model.

#### 2.2.3 Strategy for developing component sub-models required by the REFLUX study

**Figure 4:**
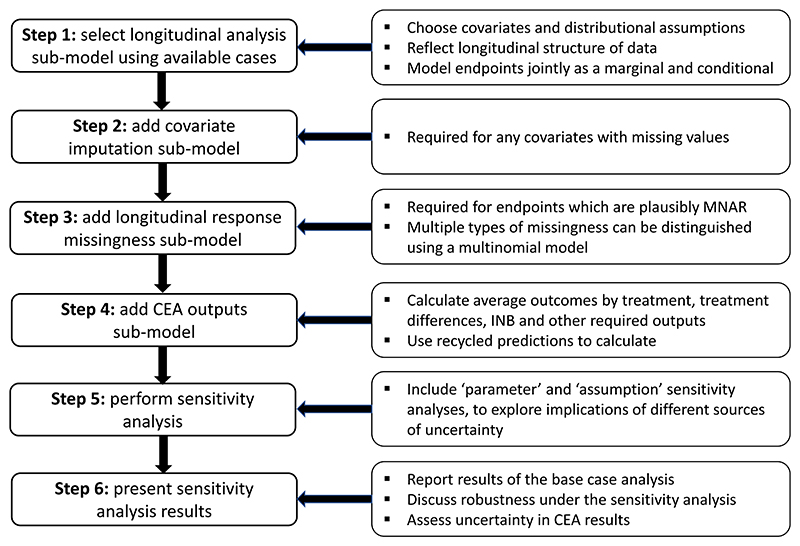
Modeling strategy for CEA based on longitudinal data. CEA, cost-effectiveness analysis [Colour figure can be viewed at wileyonlinelibrary.com]

We now draw on the REFLUX case study to exemplify the steps required to build a Bayesian selection model with appropriate complexity for CEA based on data with longitudinal structure, and how to explore the impact of different model choices on the results. Figure 4 provides an overview.

### 2.3 Step 1: select longitudinal analysis sub-model using complete cases

To further simplify the model building task, initially we work with each endpoint separately using available cases (172 for HRQoL and 191 for costs), and then assemble into a single analysis model. We consider three distributional assumptions for the HRQoL and cost endpoints: normal, gamma, and hurdle models (Grieve et al., 2010). Other options for HRQoL, such as a scaled beta or mixture models (Basu & Manca, 2012; Hernandez-Alava et al., 2012), could be included if exploratory data plots suggest that these are more appropriate. As LS is a “one-off” intervention whereas MM is an ongoing treatment strategy the trajectory of HRQoL over time may differ, and so each analysis model is parameterised separately according to treatment arm. However, to simplify notation, we suppress the treatment subscript “*tr*” in the model descriptions that follow.

The level of cross-overs rises substantially when individuals with partially observed data are also considered. Overall, 67 (37.6%) patients randomized to LS did not receive surgery, and 10 (5.6%) patients randomized to MM crossed over to receive surgery. To recognize that the distribution of costs and HRQoL reflected treatment received (cf. Figure 2d, which shows patients with total costs below £2000, who did not receive surgery), we analyze data from patients according to the treatment they actually received, but to address the decision problem of interest, we use the predictions from this analysis for patients as randomized, in line with an intention-to-treat (ITT) analysis (see Section 2.6).

#### 2.3.1 HRQoL analysis model

The REFLUX CEA required the study to report effect of randomized treatment on HRQoL each year, and then over the 5-year follow-up. We therefore chose to model HRQoL at each timepoint rather than aggregated. For simplicity, we initially ignored the multi-level structure in the data and fitted a model with each distributional assumption under consideration, incorporating the minimization covariates (age, BMI, and sex), baseline HRQoL, and time fixed effects.

As the gamma distribution is restricted to positive values, we used HRQoL decrement (1 − HRQoL + *ϵ*, where *ϵ* = 0.0001 ensured positivity) as the health outcome for all models. Accordingly, for the hurdle option, we specified the hurdle at 0 and a gamma model for the non-zeros.

Next we added normally distributed patient random intercepts to each model to account for the multi-level structure. For the hurdle model, these were incorporated into the non-zeros part of the model and not the hurdle. The normal and hurdle models ran successfully, but convergence problems were encountered for the gamma model suggesting data and model incompatibility.

Overall fit can be compared between models by using the deviance information criteria (DIC) proposed by Spiegelhalter et al. (2002), with lower values suggesting a better fit. However, the DIC automatically produced by JAGS for the hurdle model is not directly comparable with the other models because the hurdle is also modeled. The DIC showed that the random effects improved the fit of both the normal and the hurdle models. We also examined specific aspects of model fit using residuals plots and posterior predictions (see Text S1 for examples).

Based on model fit, and as suggested by the exploratory data plots which show clear spikes at 1 in the HRQoL data (Figure 2), we chose the hurdle model with patient random intercepts for both treatments. Separate for each treatment arm, the full specification is as follows:

(1) $$\begin{array}{cc} h_{it} & \sim \;\text{Bernoulli}\left(p_{it}\right)\\ \text{logit}\left(p_{it}\right) & =\;\gamma_{t}+\omega_{0}bq_{i}+\omega_{1}bmi_{i}+\omega_{2}age_{i}+\omega_{3}sex_{i}\\ q_{it}|\left(h_{it}=1\right) & =\;0\\ q_{it}|\left(h_{it}=0\right) & \sim \;\text{Gamma}\left(shape.q,shape.q/\mu .q_{it}\right)\\ \text{log}\left(\mu .q_{it}\right) & =\;\alpha_{t}+\theta_{i}+\beta_{0}bq_{i}+\beta_{1}bmi_{i}+\beta_{2}age_{i}+\beta_{3}sex_{i}\\ \theta_{i} & \sim \;\text{Normal}\left(\theta .\mu ,\theta .\sigma^{2}\right) \end{array}$$

where *q*_*it*_ is the HRQoL decrement for patient *i* at time *t, bq*_*i*_ denotes baseline HRQoL decrement for patient *i* and *θ*_*i*_ are patient random intercepts. Minimally informative priors are placed on the unknown parameters (see Appendix for details).

#### 2.3.2 Cost analysis model

For the REFLUX study, like many CEA, interest is more on the effect of treatment assignment on costs over the full-time horizon, rather than for each year. We therefore chose to model costs at the aggregate 5-year level, allowing us to demonstrate an alternative way of incorporating partially observed values. According to DIC, our chosen model is a gamma for both treatments, including the covariates BMI, age, and sex.

#### 2.3.3 Joint analysis model for HRQoL and total costs

We now combine the component sub-models into the proposed joint model. For each treatment arm, we specify the conditional cost model as follows:

(2) $$\begin{array}{cc} c_{i} & \sim \;\text{Gamma}\left(shape.c,\;shape.c/v.c_{i}\right)\\ \text{log}\left(v.c_{i}\right) & =\;\zeta_{0}+\zeta_{1}bmi_{i}+\zeta_{2}age_{i}+\zeta_{3}sex_{i}+\xi \left(Q_{i}-\mu .Q_{i}\right) \end{array}$$

where *c*_*i*_ is the aggregated 5-year costs in GBP for patient *i*. We have specified the second parameter of the gamma function (rate) in terms of the shape parameter and the conditional 5-year costs mean for individual *i, v*.*c*_*i*_. *Q*_*i*_ and *μ.Q*_*i*_ are the estimated 5-year QALYs and estimated 5-year QALY mean, respectively, for individual *i*. See Appendix for prior and other implementation details. The HRQoLs at each timepoint are combined into 5-year QALYs by linear interpolation, according to the “area under the curve” method, as follows:

(3) $$Q_{i}=0.5\left(1-q_{i1}\right)+0.875\left(1-q_{i2}\right)+\sum_{t=3}^{5}\left(1-q_{it}\right)+0.5\left(1-q_{i6}\right)$$

Similarly, *μ.Q*_*i*_ can be estimated by combining predicted values of *q*_*i*_ at each timepoint (*pred.q*_*it*_), where

(4) $$\begin{array}{l} pred.h_{it}\;\sim \;\text{Bernoulli}\left(p_{it}\right)\\ pred.q_{it}\;=\;\left(1-pred.h_{it}\right)\times \mu .q_{it} \end{array}$$

The marginal costs can be recovered using

(5) $$\mu .c_{i}=\text{exp}\left(\zeta_{0}+\zeta_{1}bmi_{i}+\zeta_{2}age_{i}+\zeta_{3}sex_{i}\right)$$

So far, we have only fitted this analysis model to patients with available data. Without making any changes, we can also include data from patients with partially observed responses provided their covariates are fully observed. For patients with HRQoL at some but not all timepoints, the specification of the disaggregated HRQoL model directly incorporates the values from observed timepoints and imputes the values that are missing. To incorporate partially observed cost information, we place a lower limit (calculated as the sum of the observed costs) on the gamma distribution for the missing aggregate 5-year costs. Without the addition of a response missingness model, the missing values are drawn from the posterior distribution assuming MAR.

### 2.4 Step 2: add covariate imputation sub-model

An analysis model will run with missing responses, but not with missing covariates. So, the next step is to specify a co-variate imputation model to impute any missing covariates. For REFLUX, BMI, age, and sex are all fully observed, but baseline HRQoL has 13 (3.6%) missing values. Given the low level of missingness, we model baseline HRQoL decrement using a Gamma distribution, that is,

(6) $$bq_{i}\sim \text{Gamma}(shape.bq,rate.bq)T(,1.2)$$

where *T* () imposes an upper bound of 1.2 on the HRQoL decrements to restrict the imputations to viable values.

### 2.5 Step 3: add longitudinal response missingness sub-model

For REFLUX, it seems likely that the probability of a patient providing their HRQoL at a particular timepoint is related to their health status at that time, so we add a heath outcome missingness model to explore different MNAR assumptions. By contrast, it is more reasonable to assume that costs are MAR as missingness is more likely to reflect administrative reasons (e.g., missing case notes) rather than a patient’s unobserved health status, and so we do not specify a cost outcome missingness model. If investigators consider that costs may also be MNAR, the response missingness model can be extended to encompass both CEA endpoints (Figure 3).

When there is a single type of missingness, a response missingness model can be specified as a logistic model for a binary missing value indicator, *m*_*i*_ (0 = observed, 1 = missing) for individual *i*. However, as with REFLUX, typically both interim missingness and loss to follow-up occur in longitudinal studies. To distinguish between multiple types of missingness, this model can be extended by specifying a multinomial logistic model for a categorical missing value indicator.

For our illustration, we incorporate covariates (age, BMI, and sex), time fixed effects, the immediate previous HRQoL, *previous.q* (baseline HRQoL is used for the first timepoint), and change from previous HRQoL, *change.q*. It is the inclusion of the possibly unobserved *change.q* that changes the assumption about the missing HRQoL from MAR to MNAR, and provides the link with the analysis model. The extent to which the missingness mechanism is assumed to depart from MAR, is captured by the parameters of *change.q*, ***λ***. Daniels and Hogan (2008) define a *sensitivity parameter* to be a parameter that is completely non-identified by the data. ***λ*** are not sensitivity parameters in this strict sense, as their estimation draws on the parametric assumptions in the analysis model and response missingness model (Mason et al., 2012)—Daniels and Hogan (2008), Section 8.3.2, provides clear examples of how this works. Indeed, selection models cannot be factorized into identifiable and non-identifiable parts. However, estimation of ***λ*** type parameters can be difficult as it is reliant on limited information from assumptions about other parts of the model and informative priors are recommended (Mason et al., 2012). Therefore, we recommend giving ***λ*** point priors and explore the sensitivity of the CEA outputs to different values.

As in the analysis model, all the parameters are allowed to differ by treatment arm. Consequently, point priors are required for four *λ* parameters: MM arm interim missing, MM arm loss to follow-up, LS arm interim missing, and LS arm loss to follow-up, with the choice informed by substantive knowledge about each intervention.

Setting *m*_*it*_ to be a three-category missing value indicator for *q*_*it*_ for patient *i* at time *t* (1 = observed, 2 = interim missing, 3 = loss to follow-up), the full specification of this multinomial logistic sub-model is as follows (suppressing the treatment subscript, *tr*, to simplify notation):

(7) $$\begin{array}{cc} {\text{count}}_{it} & \sim \;\text{Multinomial}(s_{it},1)\\ \phi_{it,1} & =\;1\\ \text{log}(\phi_{it,r}) & =\;\kappa 0_{t,r}+\kappa_{r1}bmi_{i}+\kappa_{r2}age_{i}+\kappa_{r3}sex_{i}+\kappa_{r4}previous.q_{it}+\lambda_{r}change.q_{it};\text{r}=2,3\\ s_{it,r} & =\;\frac{\phi_{it,r}}{\sum_{z=1}^{3}\phi_{it,z}} \end{array}$$

where *r* indicates the missingness category, and ***count*** is a vector with *count*_*it*,*r*_ set to 1 if *m*_*it*,*r* =_ *r* and 0 otherwise. See Appendix for prior specifications. Since this sub-model is dependent on partially observed covariates, it also links with the covariate imputation model.

### 2.6 Step 4: add CEA outputs sub-model

We report incremental cost-effectiveness using the incremental net monetary benefit (INB). The Bayesian approach allows the INB to be calculated from the posterior distribution of the parameter estimates through specifying a set of equations, which we call the CEA outputs sub-model (see Appendix for details). The uncertainty from estimating each sub-model is, therefore, propagated through to the posterior distribution of the INB, and can be encapsulated in the credible intervals around the INB estimates and other metrics such as the cost-effectiveness acceptability curve.

We use the method of *recycled predictions* which can accommodate GLMs with non-linear link functions in predicting the incremental effects (Basu & Manca, 2012; Glick et al., 2007). This method uses the fitted model to predict incremental effects using only the baseline covariates of the patients randomised into the trial, and proceeds as follows:

1. Use patient-level baseline covariates to predict outcome for all patients, assuming they are randomized to a particular treatment arm, for example usual care.
2. Analogously, predict outcome for all patients, assuming they are randomized to new treatment.
3. Calculate difference between the outcomes predicted in points 1 and 2 for each individual.
4. Incremental effects = mean of differences calculated in point 3.

To perform an ITT analysis, we predict the outcomes for cross-over patients according to the treatment they received in the trial for both points 1 and 2. Accordingly, randomization does not change their predictions, but it does tell us how to average them to obtain the ITT CEA estimate. This approach relies on a model that accurately captures the key features of the data, raising the importance of model choice.

### 2.7 Step 5: perform sensitivity analysis

To illustrate, we investigate the eight sensitivity scenarios shown in Table 1. Here, point priors for the selection parameters *λ*_*r*_ in Equation (7) are: (i) “positive MNAR selection,” a value of 0.69 = log(2), which encodes an assumption of a twofold increase in the probability of being missing for a change of 1 unit on the HRQoL scale (conditional on other variables in the selection model); (ii) MAR, corresponding to a value of zero, and (iii) “negative MNAR selection,” a value of −0.69 = log(1/2). Relative to MAR, “positive MNAR” selection leads to higher imputations for the missing components of HRQoL, and “negative MNAR” leads to lower imputations. We have chosen a twofold increase because, based on our experience, this is at the limits of plausibility. For a “live” trial, we recommend consulting experts familiar with the disease, patient population and treatments.

In Scenarios 1 and 2, for each treatment the two types of missingness are assumed to be caused by similar mechanisms, but the causes of the missingness are assumed to be different for the two treatments and have opposite effects. This type of situation has the greatest potential to lead to conclusion changing differences in the treatment effect compared with assuming MAR throughout. Scenarios 3–6 assume one type of missingness is MAR, but the other type is MNAR with the opposite effect on the two treatments. These situations will likely lead to smaller differences compared with an all MAR scenario. For Scenario 7, missingness is assumed to be associated with lower HRQoL for both missingness types and treatments, while Scenario 8 is the higher HRQoL equivalent. For Scenarios 7 and 8, any change in treatment differences will be due solely to differences in the rates of missingness between the treatments.

**Figure 5:**
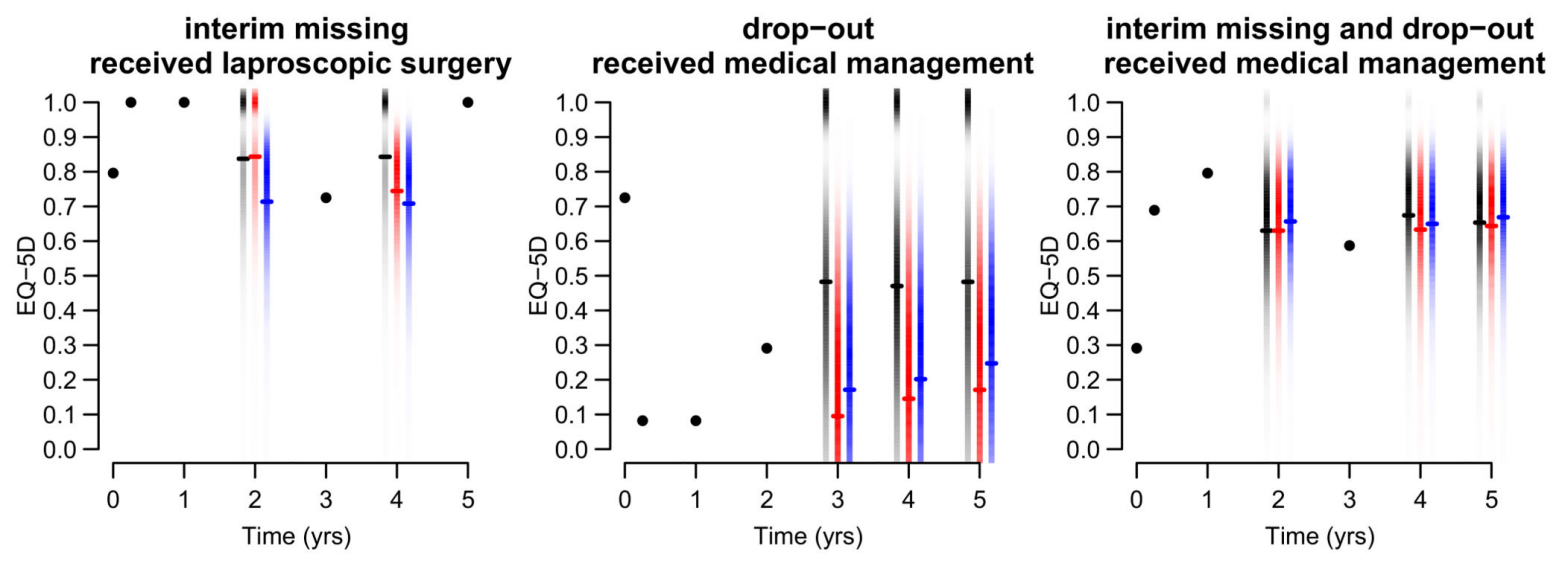
Observed and imputed data for three patients. In each panel, the closed black circles indicate observed data, and the colored strips indicate the posterior distribution of imputed data under: black: MAR; red: Table 1 Scenario 1, and blue: Table 1 Scenario 2. The probability density is represented by the color density (note the bimodal distributions for some scenarios), and the posterior mean is marked “−” [Colour figure can be viewed at wileyonlinelibrary.com]

Figure 5 shows a posterior density strip (Jackson, 2008) of imputed HRQoL data for each of three scenarios for three patients, to demonstrate the considerable uncertainty in the imputations, and to provide insight into how the posterior distributions of these imputations shift according to the missing data assumptions. The patient in the left most panel has interim missing data at 2 and 4 years; the patient in the center panel drops out from 3 years onwards and the patient in the right panel has an interim missing value at 2 years before dropping out at 4 years. The hurdle model is clearly seen in the low posterior density (light color density) just below 1. As we might expect, for the patient receiving LS, the posterior distribution from imputation under Scenario 1 (red) gives greater probability to higher values than the posterior distribution from imputation under Scenario 2 (blue). The opposite is true for the two patients receiving MM. While we might expect the posterior mean of the distribution under MAR (black) to fall between the posterior means from the two MNAR scenarios, this is not always true because these models do not contain only the sensitivity parameters; the estimated values of other model parameters will change as they are estimated to obtain best fit.

To demonstrate Step 5 of our modeling strategy, we have focused on exploring sensitivity to the choice of the selection parameters. If relevant external information is available from historical sources or experts, then further “parameter sensitivity” analysis could incorporate informative priors on other model parameters. Additionally, in studies where the choice of distribution is less obvious, we also recommend carrying out an “assumption sensitivity” to explore alternative distribution assumptions.

### 2.8 Step 6: present sensitivity analysis results

To demonstrate how our approach allows the comparison of scenarios at a disaggregate level, for analysis under complete cases only, the MAR assumption, and MNAR Scenarios 1 and 2, respectively, Table 2 shows details of mean posterior QALYs for each treatment, their difference between treatments and the probability that the difference favors LS for each year, and the sum of the QALY over the 5 years of follow-up (“5 year Total”). Comparison with the CCA analysis indicates where incorporating information from partially observed patients is particularly important. For all scenarios, there is a high probability that patients benefit from LS compared to MM throughout the 5 years, but these benefits reduce over time. The magnitude of the benefit varies between scenarios, with the benefits approximately doubling for MNAR Scenario 1 compared to MAR.

Table 3 summarizes costs and QALYs over the 5-year period for the eight MNAR scenarios, compared with MAR and CCA. Among the MNAR scenarios, MNAR1 and MNAR2 produce the highest and lowest QALY differences, respectively. The results for Scenarios MNAR3–MNAR6 reveal that in these data, the differences are driven by the assumptions about the loss to follow-up (MNAR3 is very close to MNAR1, and likewise MNAR4 to MNAR2), rather than the interim missingness (little difference between MNAR5 and MNAR6). The differences in the costs across the MNAR scenarios are small, since missing costs are always assumed to be MAR. The differences between the MAR and MNAR scenarios show the implications of the joint estimation of the analysis model and response missingness model.

**Figure 6:**
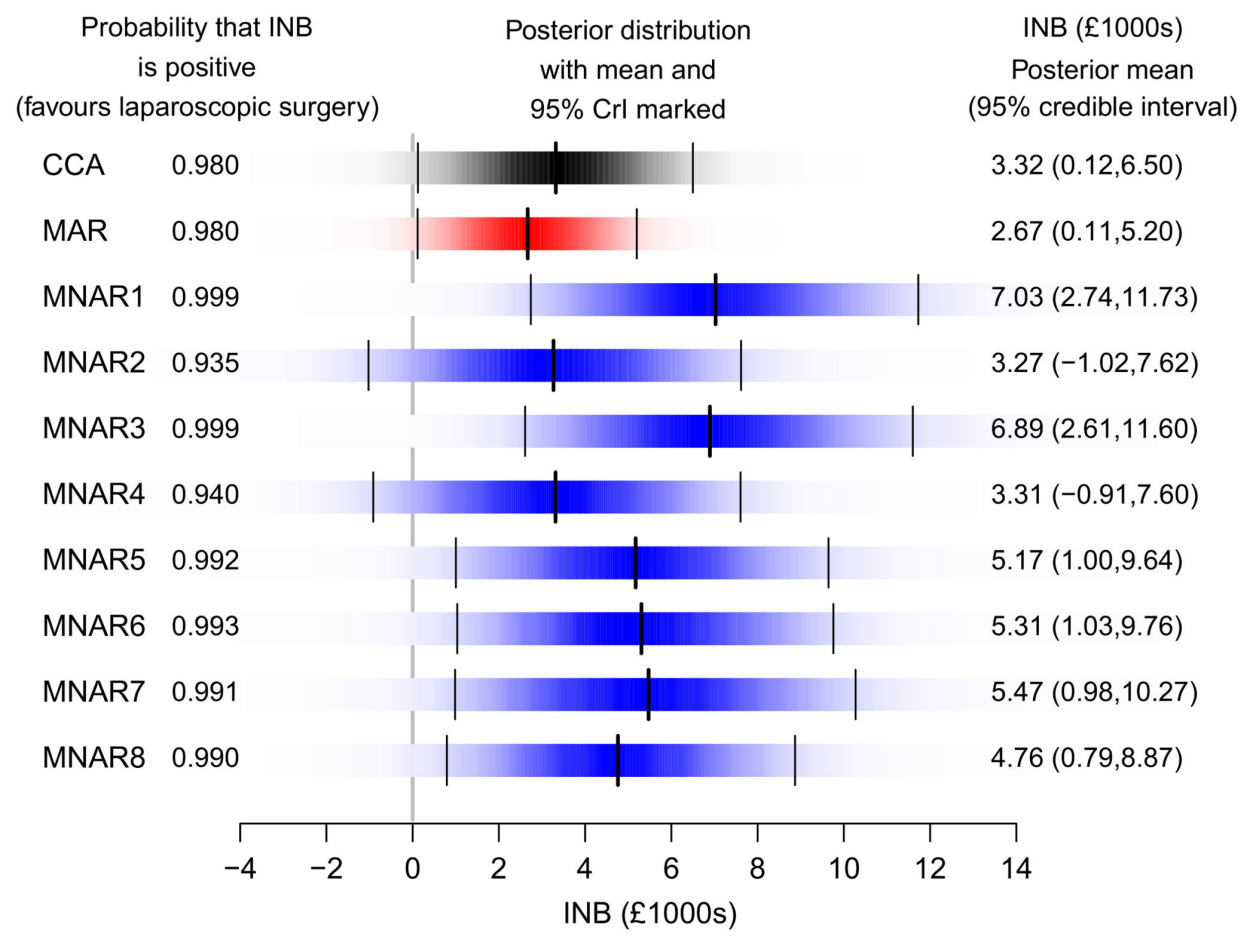
Comparison of incremental net benefit (INB). Each shaded rectangular strip shows the full posterior distribution of the incremental net benefits, valuing quality-adjusted life year gains at 20,000 GBP per quality-adjusted life year. The color density is proportional to the probability density, such that the strip is darkest at the maximum density and fades into the background at the minimum density. The posterior mean and 95% credible interval are marked. CCA, complete case analysis; CrI, credible interval; MAR, missing at random; MNAR, missing not at random [Colour figure can be viewed at wileyonlinelibrary.com]

Figure 6 shows INB, valuing quality-adjusted life year gains at 20,000 GBP per quality-adjusted life year, for the complete cases, analysis under MAR, and each of the eight MNAR scenarios in Table 1. As expected, comparing the MAR posterior distribution (shown as a density strip) and the 95% credible interval with those for CCA, reveals that incorporating extra information from the partially observed patients reduces uncertainty. However, allowing observed and unobserved HRQoL to be systematically different increases uncertainty, as shown by the increased interval widths in the MNAR scenarios, compared with both MAR and CCA.

Intuitively, we expect the estimated INB to be higher compared to the MAR analysis in MNAR scenarios with positive MNAR selection for LS and negative MNAR selection for MM (MNAR1—both types of missingness; MNAR3—loss to follow-up only; MNAR5—interim missing only), as this will increase HRQoL differences between the two treatments. Figure 6 is consistent with this expectation, and also shows that it is the loss to follow-up rather than the interim missingness which predominantly drives the increased difference. For MNAR scenarios with negative MNAR selection for LS and positive MNAR selection for MM (MNAR2—both types of missingness; MNAR4—loss to follow-up only; MNAR6—interim missing only) we expect the reverse effect, but although each of these three scenarios shifts the INB posterior density to the left of its MNAR counterpart, their posterior means are higher than for MAR. This is because, as discussed in Section 2.7, these models do not contain pure sensitivity parameters, and there will be some balancing out as the estimated values of other model parameters are adjusted to obtain best fit. For Scenarios MNAR7 and MNAR8, the MNAR selection is in the same direction for both LS and MM, so given that the proportion of missing HRQoL values is reasonably balanced across treatment arm, we expect the INB posterior means to be similar to MAR. However, consistent with the results from other MNAR scenarios, these are higher due to model fitting adjustments in other parameters.

In six of the MNAR scenarios, the probability that INB is positive is at least 99%, strongly suggesting that surgery is cost-effective. However, there is some sensitivity when missing values for patients receiving surgery are assumed lower than for other patients (MNAR2 and MNAR4), but the probabilities are still over 90% providing confidence that the findings in the primary analysis are relatively robust. Interest would likely focus on the plausibility of these scenarios in a policy decision discussion, and in a “live” CEA, further sensitivity analysis would probe these scenarios.

## 3 Discussion

This paper has developed and illustrated Bayesian selection models for CEA with informative missing data within longitudinal studies. This approach can be applied to undertake sensitivity analyses that make clearly defined, transparent assumptions. These flexible models allow the assumed missing data mechanism to differ by treatment strategy, but also according to whether the missing data reflects loss to follow-up or interim missing values. The approach also addresses typical challenges arising in CEA, such as the need to jointly model costs and outcomes, to handle non-normal distributions, and to accommodate non-compliance with the treatment assigned.

We illustrate how this approach can improve the interpretation of a study’s results, by revisiting and extending the previous analyses of the REFLUX trial (Grant et al., 2013). In the original analysis, the authors did consider that data may be MNAR, specifically assuming that HRQoL may be lower in (a) all patients with missing data and (b) those randomized to surgery who had missing data. The authors concluded that the overall conclusion, that surgery was cost-effective, was somewhat sensitive to assuming lower HRQoL in the surgery arm alone. Our methodology permits a re-analysis with a more extensive range of MNAR scenarios (8), and finds that while the results are slightly more sensitive to assumptions about “loss to follow-up” versus “interim missingness”, the conclusion that surgery is more cost-effective is robust to a wide range of alternative assumptions about the missing data mechanism. This approach can be applied directly in future studies to consider the impact of alternative missing data mechanisms on their conclusion, and in other settings, it may also be useful to distinguish the impact of “loss to follow-up” from “interim missingness”. For example, the framework can be particularly useful in studies with differential loss to follow-up according to the comparison group, as might occur in evaluating a new versus old treatment for metastatic cancer. Alternatively, the approach may accommodate greater levels of interim missingness if the new treatment has a higher incidence of side effects, versus the standard of care. In these settings, this approach provides a framework for assessing the importance of alternative, realistic assumptions about the level of HRQoL for those patients with missing data, and the potential impact on the study’s conclusions.

The proposed Bayesian selection models have important advantages compared to sensitivity analysis strategies in CEA that use pattern mixture models (Faria et al., 2014; Gabrio et al., 2020; Leurent et al., 2020; Mason et al., 2018), and build on Bayesian approaches to CEA that use RCT data (Baio, 2013; Gabrio et al., 2020; Lambert et al., 2008) and related research using Bayesian models to estimate HRQoL (Kharroubi et al., 2005, 2015, 2018). Practical advantages over pattern mixture models include (i) adopting the selection model approach enables simple conditional models to be added at each step, with some sub-models “discretionary” according to the setting; (ii) the approach can distinguish between “loss to follow-up” and “interim missingness” patterns by specifying a multinomial missing data model; (iii) the missing data and endpoint models can easily accommodate the longitudinal structure of the data, and (iv) the selection model approach requires relatively few sensitivity parameters, whereas the pattern mixture approach would require a distinct subset of models for each missing data pattern, implying many models within typical longitudinal settings. The fully Bayesian approach to selection modeling allows the uncertainty associated with the missing data to be fully propagated through the whole model, and is reflected in the final estimates of incremental cost-effectiveness.

Our example, the REFLUX study, is typical of many studies both in terms of the distribution of the costs and effects, and the longitudinal follow-up with interim missing data and loss to follow-up. Therefore, our approach has wide applicability. The breadth of applicability can be further expanded by noting that the components can be modified for CEA with different features. For example, the analyst can easily change the specification (e.g., distributions) of the models for analyzing HRQoL, for example, to incorporate beta-type models (Basu & Manca, 2012), or mixture models (Hernandez-Alava et al., 2012), or more flexible approaches to model cost data (Mihaylova et al., 2011). Also, often studies will have clinical outcomes that are correlated with the QALYs, costs, or missingness. These outcomes can be incorporated into the relevant sub-model, in the same way that we have incorporated baseline covariates, to improve the robustness of the missingness adjustments. While we focus on addressing MNAR in the health outcome, the missingness model could be developed for costs (or both endpoints). To encourage the uptake of the proposed methods, and help future studies tailor them to their needs, we provide accompanying software code to implement these models in R and JAGS (see Text S1).

Nevertheless, there are some limitations to the proposed implementation of the approach, which motivate areas for further research. First, we obtain the metrics of interest with the method of recycled predictions, in which the model predicts each endpoint for all patients for each treatment alternative. The potential drawback of this approach is in assuming the endpoint model is correctly specified. Here, the gamma-hurdle model fitted the observed data relatively well, but in other settings, it may be necessary to consider whether the results are robust to a wider set of model choices, or use observed outcome data (QALY, cost) whenever this is available. Second, the selection models were developed following publication of the primary results. Typically, we wish to pre-specify the model as part of the health economics analysis plan (Thorn et al., 2020). For selection models this requires that the analyst specifies plausible values for the sensitivity parameters a priori. A natural approach would be to elicit plausible values from experts, building on the elicitation approaches developed in Mason et al. (2018). We believe this is more sensible than the “tipping point” approach, where parameters are typically moved from the base case (typically MAR) scenario until “conclusions change” and then a posterior judgment is made as to the plausibility of these relatively extreme scenarios. Thirdly, while the REFLUX study exemplified many concerns typical in CEA that use longitudinal data, it focuses on continuous endpoints, and did not consider time to event measures such as survival time.

The framework proposed can accommodate survival outcomes but would require substantial changes in the ways the models are parameterized. Here, the response missingness model would be replaced by a model for the time to drop out, allowing informative censoring. These models can capture key features of the observed data, but would need to make plausible predictions for the period beyond the observed data (Baio, 2020; Guyot et al., 2017; Rutherford et al., 2020). More generally, our proposed modeling strategy can be used for other non-continuous types of endpoints, but the model specification would require some adaptation.

In conclusion, this Bayesian selection modeling approach, has both the flexibility and robustness to be pre-specified for the majority of CEA analyses that use RCTs with longitudinal data. We provide annotated code to support its application in future studies.

## Supplementary Material

Appendix

## Figures and Tables

**Table 1:** Eight MNAR sensitivity scenarios

	Medical management			Laparoscopic surgery	
	Interim missing	Loss to follow-up		Interim missing	Loss to follow-up
Scenario 1:	↓	↓		↑	↑
Scenario 2:	↑	↑		↓	↓
Scenario 3:	—	↓		—	↑
Scenario 4:	—	↑		—	↓
Scenario 5:	↓	—		↑	—
Scenario 6:	↑	—		↓	—
Scenario 7:	↓	↓		↓	↓
Scenario 8:	↑	↑		↑	↑

*Note*: “↑” positive MNAR selection—imputed values higher than MAR; “—” MAR imputation; “↓” negative MNAR selection—imputed values lower than MAR. Abbreviations: MAR, missing at random; MNAR, missing not at random.

**Table 2:** QALY summary by year: CCA, MAR, and two MNAR scenarios

	Mean LS^a^	Mean MM^a^	Difference^a^	Prob^b^
CCA				
Year 1	0.80 (0.77,0.83)	0.72 (0.69,0.75)	0.08 (0.04,0.12)	1.000
Year 2	0.77 (0.73,0.80)	0.71 (0.67,0.74)	0.06 (0.01,0.10)	0.996
Year 3	0.75 (0.72,0.78)	0.71 (0.68,0.74)	0.04 (0.00,0.08)	0.976
Year 4	0.74 (0.70,0.76)	0.69 (0.67,0.72)	0.04 (0.00,0.08)	0.984
Year 5	0.70 (0.67,0.73)	0.67 (0.64,0.69)	0.04 (0.00,0.07)	0.962
5 year total	3.76 (3.64,3.87)	3.50 (3.38,3.61)	0.26 (0.10,0.41)	0.999
MAR				
Year 1	0.78 (0.75,0.81)	0.70 (0.67,0.72)	0.06 (0.03,0.09)	1.000
Year 2	0.75 (0.72,0.78)	0.68 (0.65,0.71)	0.05 (0.02,0.09)	0.999
Year 3	0.73 (0.70,0.76)	0.68 (0.65,0.70)	0.04 (0.01,0.08)	0.993
Year 4	0.71 (0.68,0.74)	0.67 (0.64,0.69)	0.03 (0.00,0.06)	0.985
Year 5	0.68 (0.65,0.71)	0.64 (0.62,0.67)	0.03 (0.00,0.06)	0.968
5 year total	3.66 (3.54,3.77)	3.36 (3.25,3.47)	0.22 (0.10,0.35)	1.000
MNAR scenario 1^c^				
Year 1	0.73 (0.70,0.76)	0.62 (0.57,0.66)	0.11 (0.07,0.16)	1.000
Year 2	0.69 (0.65,0.72)	0.59 (0.53,0.63)	0.10 (0.05,0.16)	1.000
Year 3	0.66 (0.62,0.69)	0.58 (0.52,0.62)	0.08 (0.03,0.14)	1.000
Year 4	0.64 (0.60,0.67)	0.57 (0.52,0.61)	0.07 (0.03,0.12)	0.999
Year 5	0.62 (0.58,0.65)	0.55 (0.50,0.59)	0.07 (0.02,0.12)	0.998
5 year total	3.34 (3.18,3.47)	2.90 (2.65,3.10)	0.44 (0.23,0.68)	1.000
MNAR scenario 2^c^				
Year 1	0.73 (0.70,0.76)	0.64 (0.60,0.68)	0.08 (0.04,0.13)	1.000
Year 2	0.68 (0.63,0.71)	0.61 (0.56,0.65)	0.06 (0.01,0.12)	0.992
Year 3	0.65 (0.61,0.69)	0.60 (0.56,0.64)	0.04 (−0.01,0.09)	0.956
Year 4	0.63 (0.59,0.67)	0.60 (0.55,0.63)	0.03 (−0.01,0.08)	0.917
Year 5	0.61 (0.57,0.64)	0.58 (0.54,0.61)	0.03 (−0.02,0.08)	0.901
5 year total	3.29 (3.11,3.44)	3.03 (2.82,3.21)	0.26 (0.05,0.47)	0.991

Abbreviations: CCA, complete case analysis; LS, laparoscopic surgery; MAR, missing at random; MNAR, missing not at random; MM, medical management.

a Posterior mean (95% credible interval).

b Probability favors LS.

c MNAR scenarios as defined in Table 1.

**Table 3:** Five-year summary: CCA, MAR, and MNAR sensitivity analyses

	QALYs				Costs (£1000s)			
	Mean LS^a^	Mean MM^a^	Difference^a^		Mean LS^a^	Mean MM^a^	Difference^a^
CCA	3.76 (3.64,3.87)	3.50 (3.38,3.61)	0.26 (0.10,0.41)		3.33 (3.06,3.63)	1.41 (1.03,2.05)	1.92 (1.24,2.41)
MAR	3.66 (3.54,3.77)	3.36 (3.25,3.47)	0.22 (0.10,0.35)		3.53 (3.27,3.82)	1.21 (0.93,1.60)	1.83 (1.46,2.16)
MNAR1	3.34 (3.18,3.47)	2.90 (2.65,3.10)	0.44 (0.23,0.68)		3.21 (2.97,3.47)	1.34 (1.05,1.74)	1.87 (1.49,2.21)
MNAR2	3.29 (3.11,3.44)	3.03 (2.82,3.21)	0.26 (0.05,0.47)		3.22 (2.98,3.49)	1.34 (1.05,1.74)	1.88 (1.50,2.23)
MNAR3	3.34 (3.18,3.47)	2.90 (2.65,3.10)	0.44 (0.23,0.67)		3.21 (2.98,3.47)	1.34 (1.05,1.75)	1.87 (1.48,2.21)
MNAR4	3.29 (3.12,3.44)	3.03 (2.83,3.21)	0.26 (0.05,0.47)		3.22 (2.98,3.49)	1.34 (1.05,1.74)	1.88 (1.50,2.23)
MNAR5	3.32 (3.15,3.46)	2.97 (2.74,3.15)	0.35 (0.15,0.58)		3.21 (2.98,3.47)	1.34 (1.05,1.73)	1.87 (1.49,2.21)
MNAR6	3.32 (3.15,3.46)	2.96 (2.73,3.15)	0.36 (0.15,0.58)		3.21 (2.97,3.48)	1.34 (1.05,1.73)	1.87 (1.49,2.22)
MNAR7	3.26 (3.08,3.41)	2.89 (2.65,3.10)	0.37 (0.14,0.61)		3.22 (2.98,3.48)	1.34 (1.05,1.75)	1.87 (1.48,2.21)
MNAR8	3.37 (3.22,3.50)	3.04 (2.82,3.22)	0.33 (0.13,0.53)		3.21 (2.97,3.46)	1.34 (1.04,1.72)	1.87 (1.50,2.21)

Abbreviations: CCA, complete case analysis; LS, laparoscopic surgery; MAR, missing at random; MM, medical management; MNAR, missing not at random.

a Posterior mean (95% credible interval).

## Data Availability

Data sharing is not applicable to this article as no new data were created or analyzed in this study.
