# Supplementary material for: Flexible Bayesian longitudinal models for cost-effectiveness analyses with informative missing data
Source: Health Econ. Author manuscript; Available in PMC 2024 Dec 9. (PMC7617114; doi:10.1002/hec.4408)
Supplement: Appendix [file EMS176926-supplement-Appendix.pdf]

SUPPORTING INFORMATION

Additional supporting information may be found online in the Supporting Information section at the end of the article.

APPENDIX

A1 MODEL IMPLEMENTATION

We used the statistical software R (R Core Team, 2020) for pre-processing the trial data and post-processing the posterior samples, which were generated using the JAGS software (Plummer, 2003), called via the R package runjags (Denwood, 2016). All the models were run with two chains initialised using diffuse starting values to produce a sample of 10000 after convergence for posterior inference, providing an effective sample size of at least 3000 for the quantities of interest (thinning set to 10). Convergence is assumed if the potential scale reduction factor of the Gelman-Rubin statistic (Gelman & Rubin, 1992) is less than 1.05 for individual model parameters and a visual inspection of the trace plot for each parameter is satisfactory.

To implement the hurdle model for analysing HRQoL, we created a zero value indicator, h, set to 1 if HRQoL = 1 (i.e. HRQoL decrement is 0) and 0 otherwise. We explored different parameterisations of the gamma model for prior specification, and recommend using the shape and mean as this reduces the correlation between parameters compared to other options (e.g. using the mean and sd led to very high correlation between the mean intercept and sd). Also, in line with the usual recommendations for fitting Bayesian models, we scaled and centered all covariates, including binary variables. An upper limit of 1.6 was imposed on the gamma distributions, consistent with the range of legitimate values for EQ-5D.

A1.1 Selection of priors

We generally select minimally informative priors, the exception being the 'sensitivity type' parameters in the response missingness model that control the amount of departure from MAR (Section 2.5). Following the recommendations in Lunn et al. (2013) for logistic regression models, we place logistic(0, 1) priors on the intercept and normal$(0, 1.65^2)$ priors on the other regression coefficients (normal parameterised in terms of the mean and variance). These generate approximately flat priors on the probability scale. For other regression models, the location and scale parameters are given normal$(0, 10^2)$ priors and uniform(0, 100) priors respectively. As any correlation between the endpoints is expected to be negative, we restrict the prior on the ξ parameter (Equation (2)) to negative values, normal$(0, 10^2)T(, 0)$.

A2 CEA OUTPUT EQUATIONS

Here, we provide the equations for the CEA outputs sub-model. We no longer suppress the treatment subscript, tr ($tr = 1$ denotes MM; $tr = 2$ denotes LS), and to simplify we denote the baseline covariates for patient i as vector $\boldsymbol{X}_i$.

Estimate HRQoL decrement assuming patients receive treatment 1 (MM) as follows:

$$\begin{aligned} h_{1,it} &\sim \text{Bernoulli}(p_{1,it}) \\ \text{logit}(p_{1,it}) &= \gamma_{t,tr=1} + \boldsymbol{\omega}_{tr=1}^{T}\boldsymbol{X}_i \\ \mu.q_{1,it} &= \exp(\alpha_{t,tr=1} + \theta_{1,i} + \beta_{tr=1}^{T}\boldsymbol{X}_i) \times (1 - h_{1,it}) \\ \theta_{1,i} &\sim \text{Normal}(\theta.\mu_{tr=1}, \theta.\sigma_{tr=1}^{2}). \end{aligned}$$

Estimate the HRQoL decrement assuming patients receive treatment 2 (LS) analogously.

Predict HRQoL decrement, assuming cross-over patients always receive the treatment they received during the trial

10991050, 2021, 12, Downloaded from https://onlinelibrary.wiley.com/doi/10.1002/hec.4408 by University College London UCL Library Services, Wiley Online Library on [07/06/2023]. See the Terms and Conditions (https://onlinelibrary.wiley.com/terms-and-conditions) on Wiley Online Library for rules of use; OA articles are governed by the applicable Creative Commons License

$$pred.q_{1,it} = (\mu.q_{1,it} \times (1 - MMxover_i)) + (\mu.q_{2,it} \times MMxover_i)$$
$$pred.q_{2,it} = (\mu.q_{2,it} \times (1 - LSxover_i)) + (\mu.q_{1,it} \times LSxover_i)$$

where *MMxover* is a binary indicator variable set to 1 if the patient was randomised to MM but received LS; and 0 otherwise. *LSxover* is defined analogously.

Estimate HRQoL differences: $diff.q_i = pred.q_{1,i} - pred.q_{2,i}$.

Estimate 1-year QALY differences

$$diff.Q_{iy} = \begin{cases} (0.5 \times diff.q_{iy}) + (0.375 \times diff.q_{i(y+1)}) & y = 1 \\ 0.5 \times (diff.q_{iy} + diff.q_{i(y+1)})/discount^{y-1} & y > 1. \end{cases}$$

Estimate 5-year QALY differences: $diff.Qtot_i = \sum_y diff.Q_{iy}$.

Estimate 5-year QALY increment: $Qinc = \frac{1}{N}\sum_i diff.Qtot_i$, where N is the total number of patients in the trial.

Estimate costs assuming patients receive treatment 1 (MM): $\mu.c_{1,i} = \exp(\zeta_{0,tr=1} + \boldsymbol{\zeta}^T_{tr=1}\boldsymbol{X}_i)$, and estimate costs assuming patients receive treatment 2 (LS) analogously.

Predict costs, assuming cross-over patients always receive the treatment they received during the trial

$$pred.c_{1,i} = (\mu.c_{1,i} \times (1 - MMxover_i)) + (\mu.c_{2,i} \times MMxover_i)$$
$$pred.c_{2,i} = (\mu.c_{2,i} \times (1 - LSxover_i)) + (\mu.c_{1,i} \times LSxover_i).$$

Estimate cost differences

$$diff.C_i = pred.c_{1,i} - pred.c_{2,i}$$
$$Cinc = \frac{1}{N}\sum_i diff.C_i.$$

Estimate incremental net benefits (INB), valuing quality-adjusted life year gains at 20,000GBP per quality-adjusted life year

$$INB = (20000 \times Qinc) - Cinc.$$

A3 JAGS JOINT MODEL CODE

```
# JAGS joint model of CEA for the REFLUX trial - QALYs allowed to be MNAR (selection model)
# analysis model for QALYs and Costs, 2 arms separately parameterised
#     HRQoL: hurdle at 0, with Gamma for non-zeros (priors on shape (shape.q) and mean (mu.q) coefficients)
#     Costs: gamma
#     HRQoL part includes fixed time effects, baseline HRQoL decrement (bq) and covariates (X),
#        and individual random effects in non-zeros model
#     Cost part includes covariates and is conditional on QALYs
#         and incorporates information from partially observed costs by imposing a lower bound
# covariate imputation model for baseline HRQoL (bq)
# response missingness model distinguishes 2 types of missingness using multinomial logistic model
#     all parameters vary by treatment arm
#     includes time fixed effects, covariates (X), last HRQoL observation (previous.q)
#        and change from last HRQoL observation (change.q)
# q = HRQoL decrements (1-HRQoL)
# h = 1 if q = 0; 0 if q > 0 (zero value indicator)
# cost = total costs in £1000s over 5 years
# treatment arm is indexed by tr in this model (1 = MM and 2 = LS)
# MMxover = 1 if patient randomised to MM but receives LS; 0 otherwise
# LSxover = 1 if patient randomised to LS but receives MM; 0 otherwise
```

10991050, 2021, 12, Downloaded from https://onlinelibrary.wiley.com/doi/10.1002/hec.4408 by University College London UCL Library Services, Wiley Online Library on [07/06/2023]. See the Terms and Conditions (https://onlinelibrary.wiley.com/terms-and-conditions) on Wiley Online Library for rules of use; OA articles are governed by the applicable Creative Commons License

```
# prepare data for multinomial logistic response missingness sub-model
data {
   for (i in 1:Np) { # loop through individuals
      for (t in 1:Nt) { # loop through timepoints provided patients have not already dropped out
         for (r in 1:3) {count[i,t,r] <- equals(mind[i,t],r)} # set up multinomial count
      }
   }
}

model{

   # ***** marginal model for health outcome *****

   # specify marginal hurdle sub-model for HRQoL decrements
   for (t in 1:Nt) { # 6 timepoints
      for (i in 1:Np) { # Np individuals
         h[i,t] ~ dbern(p[i,t]) # 1 indicates zeros model
         logit(p[i,t]) <- gamma[t,tr[i]] + omega0[tr[i]]*bqC[i] + inprod(omega[1:Nx,tr[i]],X[i,1:Nx])
         d[i,t] <- h[i,t]+1 # model index (1 = MM and 2 = LS)
         q[i,t] ~ dgamma(shape.q[d[i,t],tr[i]],rate.q[d[i,t],i,t])T(,1.6)
         pred.h[i,t] ~ dbern(p[i,t])
         pred.q[i,t] <- (1-pred.h[i,t])*mu.q[i,t] # prediction is 0 if h=1

         # non-zeros model
         log(mu.q[i,t]) <- alpha[t,tr[i]] + theta[i] + beta0[tr[i]]*bqC[i] + inprod(beta[1:Nx,tr[i]],X[i,])
         rate.q[1,i,t] <- shape.q[1,tr[i]]/mu.q[i,t]

         # zeros model - not used in QALY increment calculation
         rate.q[2,i,t] <- shape.q[2,tr[i]]/mu.q0

         # calculate residuals
         resid[i,t] <- q[i,t] - pred.q[i,t]

         # predict HRQoL assuming all participants have treatment 1 (MM)
         h1[i,t] ~ dbern(p1[i,t])
         logit(p1[i,t]) <- gamma[t,1] + omega0[1]*bqC[i] + inprod(omega[1:Nx,1],X[i,1:Nx])
         mu.q1[i,t] <- exp(alpha[t,1]+theta1[i]+beta0[1]*bqC[i]+inprod(beta[1:Nx,1],X[i,1:Nx])) * (1-h1[i,t])# 0 if h0=1
         # prediction assuming cross-overs receive LS
         pred.q1[i,t] <- (mu.q1[i,t] * (1-MMxover[i])) + (mu.q2[i,t] * MMxover[i])

         # predict HRQoL assuming all participants have treatment 2 (LS)
         h2[i,t] ~ dbern(p2[i,t])
         logit(p2[i,t]) <- gamma[t,2] + omega0[2]*bqC[i] + inprod(omega[1:Nx,2],X[i,1:Nx])
         mu.q2[i,t] <- exp(alpha[t,2]+theta2[i]+beta0[2]*bqC[i]+inprod(beta[1:Nx,2],X[i,1:Nx])) * (1-h2[i,t]) # 0 if h0=1
         # prediction assuming cross-overs receive MM
         pred.q2[i,t] <- (mu.q2[i,t] * (1-LSxover[i])) + (mu.q1[i,t] * LSxover[i])

         # calculate HRQoL differences
         diff.q[i,t] <- pred.q1[i,t] - pred.q2[i,t] # difference is LS-MM HRQoL (switch from HRQoL decrement)
      }
   }

   for (i in 1:Np) { # individual random effects for HRQoL marginal model
```

10991050, 2021, 12, Downloaded from https://onlinelibrary.wiley.com/doi/10.1002/hec.4408 by University College London UCL Library Services, Wiley Online Library on [07/06/2023]. See the Terms and Conditions (https://onlinelibrary.wiley.com/terms-and-conditions) on Wiley Online Library for rules of use; OA articles are governed by the applicable Creative Commons License

```
    theta[i] ~ dnorm(theta.mu[tr[i]],theta.tau[tr[i]])
    theta1[i] ~ dnorm(theta.mu[1],theta.tau[1]) # random effects for treatment 1 predictions
    theta2[i] ~ dnorm(theta.mu[2],theta.tau[2]) # random effects for treatment 2 predictions
}

# prior distributions for HRQoL marginal sub-model
for (a in 1:2) { # 2 treatment arms
    for (t in 1:Nt) {gamma[t,a] ~ dlogis(0,1)} # time fixed effects for hurdle
    omega0[a] ~ dnorm(0,0.368)
    for (i in 1:Nx) {omega[i,a] ~ dnorm(0,0.368)}
    alpha[1,a] <- 0
    for (t in 2:Nt) {alpha[t,a] ~ dnorm(0,0.01)} # time fixed effects for non-zeros model
    beta0[a] ~ dnorm(0,0.01)
    for (i in 1:Nx) {beta[i,a] ~ dnorm(0,0.01)}
    shape.q[1,a] ~ dunif(0,100)

    theta.mu[a] ~ dnorm(0,0.01) # prior on random effects mean
    theta.sigma[a] ~ dunif(0,100) # prior on random effects sd

    # node transformations
    theta.sigma2[a] <- pow(theta.sigma[a],2)
    theta.tau[a] <- 1/theta.sigma2[a]
}

# set mean and sd of zeros model to induce a spike close to 0
mu.q0 <- 0.0001
for (a in 1:2) {shape.q[2,a] <- 0.0001}

# ***** conditional model for cost outcome *****

# specify conditional gamma sub-model for costs
for (i in 1:Np) { # Np individuals
    # switch from HRQoL decrements to HRQoL to calculate QALYs
    Qtot[i] <- (0.5*(1-q[i,1])) + (0.875*(1-q[i,2])) + sum((1-q[i,3:5])) + (0.5*(1-q[i,6]))
    Qmu[i]<-(0.5*(1-pred.q[i,1]))+(0.875*(1-pred.q[i,2]))+sum((1-pred.q[i,3:5]))+(0.5*(1-pred.q[i,6]))

    # model costs conditional on QALYS
    cost[i] ~ dgamma(shape.c[tr[i]],rate.c[i])T(lower[i],)
    log(mu.c[i]) <- zeta0[tr[i]] + inprod(zeta[1:Nx,tr[i]],X[i,]) + xi[tr[i]]*(Qtot[i]-Qmu[i])
    rate.c[i] <- shape.c[tr[i]]/mu.c[i]

    # predict cost assuming all participants receive treatment 0
    mu.c1[i] <- exp(zeta0[1] + inprod(zeta[1:Nx,1],X[i,1:Nx]))
    # prediction assuming cross-overs receive LS
    pred.c1[i] <- (mu.c1[i] * (1-MMxover[i])) + (mu.c2[i] * MMxover[i])

    # predict cost assuming all participants receive treatment 1
    mu.c2[i] <- exp(zeta0[2] + inprod(zeta[1:Nx,2],X[i,1:Nx]))
    # prediction assuming cross-overs receive MM
    pred.c2[i] <- (mu.c2[i] * (1-LSxover[i])) + (mu.c1[i] * LSxover[i])

    # calculate cost differences
```

10991050, 2021, 12, Downloaded from https://onlinelibrary.wiley.com/doi/10.1002/hec.4408 by University College London UCL Library Services, Wiley Online Library on [07/06/2023]. See the Terms and Conditions (https://onlinelibrary.wiley.com/terms-and-conditions) on Wiley Online Library for rules of use; OA articles are governed by the applicable Creative Commons License

```
    Ctot.diff[i] <- pred.c2[i] - pred.c1[i]
}

# prior distributions for cost conditional sub-model
for (a in 1:2) { # 2 treatment arms
    zeta0[a] ~ dnorm(0,0.01)
    for (i in 1:Nx) {zeta[i,a] ~ dnorm(0,0.01)}
    xi[a] ~ dnorm(0,0.01)T(,0) # any correlation expected to be negative
    shape.c[a] ~ dunif(0,100)
}

# ***** covariate imputation model *****

# specify covariate imputation model for baseline HRQoL decrement
for (i in 1:Np) {
    bq[i] ~ dgamma(shape.bq,rate.bq)T(,1.2)
    # center and standardise for HRQoL marginal sub-model
    bqC[i] <- (bq[i]-mean.bq)/sd.bq
}

# prior distributions for covariate imputation model
shape.bq ~ dunif(0,100)
mu.bq ~ dunif(0,1.2)
rate.bq <- shape.bq/mu.bq

# ***** health outcome missingness model *****

# specify response missingness model for HRQoL
for (i in 1:Np) { # loop through individuals
    previous.q[i,1] <- bq[i]
    for (t in 2:Nt) {previous.q[i,t] <- q[i,t-1]}
    for (t in 1:Nt) { # loop through all timepoints
        count[i,t,1:3] ~ dmulti(m[i,t,1:3],1)
        change.q[i,t] <- q[i,t]-previous.q[i,t]
        for (r in 1:3) {
            m[i,t,r] <- phi[i,t,r]/sum(phi[i,t,])
            log(phi[i,t,r]) <- kappa0[r,t,tr[i]] + inprod(kappa[r,1:Nx,tr[i]],X[i,1:Nx])
                + kappa[r,4,tr[i]]*(previous.q[i,t]-mean.q)/sd.q + lambda[r,tr[i]]*change.q[i,t]
        }
    }
}

# prior distributions for response missingness model
for (a in 1:2) { # 2 treatment arms
    for (t in 1:Nt) {kappa0[1,t,a] <- 0}
    for (i in 1:4) {kappa[1,i,a] <- 0}
    for (r in 2:3) {
        for (t in 1:Nt) {kappa0[r,t,a] ~ dlogis(0,1)}
        for (i in 1:4) {kappa[r,i,a] ~ dnorm(0,0.01)}
    }
}

# ***** calculation of CEA outputs model *****
```

10991050, 2021, 12, Downloaded from https://onlinelibrary.wiley.com/doi/10.1002/hec.4408 by University College London UCL Library Services, Wiley Online Library on [07/06/2023]. See the Terms and Conditions (https://onlinelibrary.wiley.com/terms-and-conditions) on Wiley Online Library for rules of use; OA articles are governed by the applicable Creative Commons License

```
# calculate QALY differences over 5 year period
for (i in 1:Np) { # Np individuals
   Qaly1[i,1] <- (0.125*(1-bq[i])) + (0.5*(1-pred.q1[i,1])) + (0.375*(1-pred.q1[i,2])) # QALY for MM in year 1
   Qaly2[i,1] <- (0.125*(1-bq[i])) + (0.5*(1-pred.q2[i,1])) + (0.375*(1-pred.q2[i,2])) # QALY for LS in year 1
   Q.diff[i,1] <- (0.5*diff.q[i,1]) + (0.375*diff.q[i,2]) # QALY difference in year 1
   for (y in 2:5) { # years 2 to 5, applying discount
      Qaly1[i,y] <- 0.5 *(2-pred.q1[i,y]-pred.q1[i,y+1]) / pow(disc,y-1) # QALY for MM
      Qaly2[i,y] <- 0.5 *(2-pred.q2[i,y]-pred.q2[i,y+1]) / pow(disc,y-1) # QALY for LS
      Q.diff[i,y] <- 0.5 *(diff.q[i,y]+diff.q[i,y+1]) / pow(disc,y-1) # QALY difference
   }
   Qtot1[i] <- sum(Qaly1[i,]) # 5-year QALYs for MM
   Qtot2[i] <- sum(Qaly2[i,]) # 5-year QALYs for LS
   Qtot.diff[i] <- sum(Q.diff[i,]) # 5-year QALY difference
}

# calculate QALY increment using recycled predictions
AveQ[1] <- mean(Qtot1[])
AveQ[2] <- mean(Qtot2[])
Qinc <- mean(Qtot.diff[]) # 5-year QALY increment
p.Qinc <- step(Qinc) # probability favours LS
for (y in 1:5) { # calculate QALY increment by year
   AveQ.1yr[y,1] <- mean(Qaly1[,y])
   AveQ.1yr[y,2] <- mean(Qaly2[,y])
   Q1yr.inc[y] <- mean(Q.diff[,y]) # 1-year QALY increment
   p.Q1yr[y] <- step(Q1yr.inc[y]) # probability favours LS
}

# calculate cost increment using recycled predictions
AveC[1] <- mean(pred.c1[])*1000
AveC[2] <- mean(pred.c2[])*1000
Cinc <- mean(Ctot.diff[])*1000 # 5-year cost increment
p.Cinc <- 1-step(Cinc) # probability favours LS (Cinc positive)

# calculate incremental net benefits (INB)
for (j in 1:M) { # values of efficacy (QALY) gains
      inb[j] <- (threshold[j]*Qinc)-Cinc
      p.ce[j] <- step(inb[j]) # probability favours LS (INB positive)
}
}
```

10991050, 2021, 12, Downloaded from https://onlinelibrary.wiley.com/doi/10.1002/hec.4408 by University College London UCL Library Services, Wiley Online Library on [07/06/2023]. See the Terms and Conditions (https://onlinelibrary.wiley.com/terms-and-conditions) on Wiley Online Library for rules of use; OA articles are governed by the applicable Creative Commons License
